# Thailand special recruitment track of medical students: a series of annual cross-sectional surveys on the new graduates between 2010 and 2012

**DOI:** 10.1186/1478-4491-11-47

**Published:** 2013-09-24

**Authors:** Weerasak Putthasri, Rapeepong Suphanchaimat, Thitikorn Topothai, Thunthita Wisaijohn, Noppakun Thammatacharee, Viroj Tangcharoensathien

**Affiliations:** 1 International Health Policy Program (IHPP), Ministry of Public Health, Tivanond Road, Nonthaburi 11000, Thailand; 2 London School of Hygiene and Tropical Medicines, University of London, London, Keppel St, London WC1E 7HT, United Kingdom

**Keywords:** Physicians, Medical graduate, Medical education, Supply and distribution, Professional competence, Attitude of health personnel

## Abstract

**Background:**

Comprehensive policies for rural retention of medical doctor and other health professional, including education strategy and mandatory service, have been implemented in Thailand since the 1970s. This study compared the rural attitudes, intention to fulfil mandatory rural service and competencies between medical graduates’ from two modes of admission, normal and special tracks.

**Methods:**

Three cross-sectional, self-administered questionnaire surveys were conducted in April 2010, 2011 and 2012. The questionnaire was distributed to all new medical graduates in the annual Ministry of Public Health meeting to allocate workplaces for the 3-year mandatory service.

**Findings:**

The majority of students were recruited through the normal track (56 to 77%) from medical schools in Bangkok (56 to 66%), having mostly attended secondary schools in Bangkok. A majority of special track graduates came from secondary schools in provincial cities (76 to 79%). All three batches came from well-educated parents.

A slight difference in rural attitudes was observed between tracks. Univariable analysis found statistical associations between the intention to fulfil the 3-year obligation and special track recruitment and attributes on rural exposure. Multivariable analysis showed that graduates recruited through the special track had a 10 to 15% higher probability of fulfilling the mandatory service.

Special track graduates scored higher on four out of five competencies, notably procedural skills, but normal track graduates had higher competency on clinical knowledge in major clinical subjects.

**Conclusion:**

Since special track recruitment resulted in a higher probability of fulfilling mandatory service and competency, increasing the proportion of special track recruitment and improving the effectiveness of policies addressing physician shortage were recommended.

## Introduction

Shortage and maldistribution of the health workforce, a key component of health systems, has shown limited progress, especially among the critical shortage countries [1,2], far below the recommended threshold of 2.28 per 1,000 population [3]. Density of the health workforce contributed to health achievement [4,5]. Shortage and maldistribution are two major bottlenecks facing lower- and middle-income countries [6]. Inequitable distribution aggravates shortages in rural and hard-to-reach areas.

Empirical evidence suggests that having medical students with rural backgrounds has a positive influence on rural primary care practices and retention. In the US, the Physician Shortage Area Program demonstrated that a student’s rural background contributed to rural primary care practice and retention [7]; similar findings were reported from Canada and Japan [8-10]. Studies in Australia and South Africa confirmed that early rural exposure supported students gaining knowledge and skills necessary for future work in rural areas [11-14].

Five out of sixteen interventions to improve health workforce retention in rural areas, as recommended by the World Health Organization, were education strategies such as recruiting students from rural backgrounds, locating health professional schools outside major cities and clinical rotations in rural areas during studies [15]. In Thailand, a mix of these policy interventions were gradually introduced since the 1970s [16-18].

There are two distinct modes of admission for medical students in Thailand. Under the normal track, any grade-12 student can apply to sit the national entrance examination; based on competency, they are recruited to study medicine for 6 years (1 year basic science, 2 years preclinical and 3 years clinical) in one of the 19 faculties of medicines operating under the Ministry of Education. Prior to the national entrance examination, some faculties convene their own recruitment (so-called direct admission) based on competitiveness and competency, judged by their institution-specific examination papers.

Under the special track, two ongoing national programs are the ‘Collaborative Project to Increase Production of Rural Doctors (CPIRD)’ launched in 1995 and ‘One District One Doctor (ODOD)’ launched in 2005. Any grade-12 students residing in a provincial area (for CPIRD) or in a non-provincial city (for ODOD) are eligible to sit an examination jointly convened by all 19 faculties of medicine, using a single examination paper. Students are then recruited based on competency. Students on the CPIRD and ODOD tracks take the first 3 years jointly with normal track students in universities, but during their 3 clinical years they are trained by clinical staff in the 34 accredited regional and provincial hospitals of the Ministry of Public Health (MOPH), affiliated with relevant faculties of medicine [19,20]. Their diplomas are granted by their individual university, not the affiliated MOPH institutes.

Medical students from all tracks are exposed to rural services in their clinical years and are subject to a national license examination to obtain their medical license. Graduates from all tracks are liable for mandatory service (3 years for normal track students and CPIRD, and 12 years for ODOD), with a financial penalty of US$ 13,000 for non-adherence by normal track and CPIRD graduates, and five times higher (US$ 65,000) for ODOD graduates. The difference in choosing workplace between normal track and CPIRD/ODOD is that, upon graduation, students in CPIRD/ODOD track are obliged to work in certain severe shortage provinces where allocations are mostly their hometown or neighboring districts/provinces while the choice of workplace for normal track graduates are more flexible, depending on their own choice given the availability of vacant posts [20].

Despite the implementation of a national program to solve the health worker shortage, there has not been a comprehensive study to assess the impact of the special track in relation to normal track recruitment. This study seeks to fill this knowledge gap by comparing CPIRD/ODOD with normal track graduates on their rural attitudes, intention to fulfil the 3-year mandatory service, and competency.

## Methods

### Study design

Three consecutive, cross-sectional surveys were conducted with all new medical graduates who gathered at the meeting convened by the MOPH to choose hospitals for their mandatory rural services on 2 April 2010, 1 April 2011, and 1 April 2012, respectively.

### Population and sample size

The target population was all the 1,545, 1,479 and 1,679 new medical graduates in 2010, 2011, and 2012 who gathered in the meeting convened by MOPH to choose their work place. The questionnaire was distributed to all these new graduates along with their registration documents. Having given verbal consent, a total of 576 (37.2%), 872 (59.0%) and 754 (44.9%) graduates from each year anonymously responded to the questionnaire. Confidentiality of information was strictly kept according to the standard operating procedure of health science research. The National Ethical Review Committee waived ethical clearance for this study.

### Questionnaire design

The questionnaire was developed from a consultative meeting in the MOPH. Content validity was checked by canvassing the opinions of senior officers in the MOPH. A reliability test was performed through a pilot test on the final-year medical students in KhonKaen University (Cronbach’s alpha = 0.64; considered acceptable reliability). There were four parts in the questionnaire: (1) demographics of graduates including age, gender, mode of admission to medical education(normal admission track through competitive national entrance examination or direct admission, and special tracks under CPIRD and ODOD), location of their medical school (in Bangkok or outside), location of residence for the ages 1 to 15 years, location of current residence, location of secondary school, and education profile of parents; (2) attitudes toward rural practice measured by level of attitude using the Likert scale, ranging from one (least positive) to five (most positive) arranged in five questions indicating ‘peers in rural settings are supportive’, ‘working in rural areas provides greater opportunity in meeting professional colleagues’, ‘amenities and facilities are available in rural areas’, ‘people in rural areas are generous and friendly’, and ‘working in rural areas can maintain close contact with friends and families; (3) intention to complete the 3-year mandatory service and the plans thereafter; and (4) confidence in medical and public health competency using fourteen questions and self assessment using the Likert score ranging from one (least confident) to five (most confident). All the four parts were arranged in a single attachment.

The fourteen competency questions cover: (1) public health; (2) health administration; (3) communication; (4) inter-professional collaboration; (5) internal medicine care; (6) obstetric and gynecologic care; (7) pediatric care; (8) surgical care; (9) general disease care; (10) difficult labor management; (11) patient referral management; (12) overall clinical competency; (13) clinical knowledge; and (14) clinical skill. The questionnaire was tested on the content validity, then redesigned and finalized. Completed questionnaires were dropped in a collecting box anonymously.

### Data analysis

Analyses were performed using Stata Version 11 (STATA Corporation, College Station, TX, USA). The analysis consisted of four parts: (1) descriptive statistics were used to understand the socio-demographic profiles of graduates and their parents, and Pearson’s Chi-square test was used to explain the association between modes of admission and location of secondary school; (2) average rural attitude scores were compared between two modes of admission; (3) Pearson’s Chi-square was used to explain association between various attributes and the intention to fulfil the 3-year mandatory service; furthermore, multivariable logistic regression with marginal effects was conducted to determine the association between the intentions to fulfil the 3-year obligations and the significant attributes identified by the univariable analysis; and (4) a composite competency score was produced using exploratory factor analysis of the fourteen competency questions. Principal component analysis was applied to identify factors which addressed around at least 75% of variance. Varimax rotation was executed to group the fourteen questions into each factor. The composite score of each factor was calculated using Bartlett’s formula [21]. The competency composite score was assessed against the mode of admission.

## Results

### Profile of samples

Across the three batches, graduates were on average 24 years old and more were female than male. It was noted that the majority of graduates came from normal track recruitment and graduated from medical schools located in Bangkok (Table 1). Opportunities for students from secondary schools located outside provincial cities to enrol in medical education was small, around 11.5 to 15.0% of total graduates, while a majority of graduates were students from secondary schools located either in Bangkok (33.0 to 53.2%) or in a provincial city (35.3 to 52.0%). A majority of all three batches of medical graduates came from well-educated parents; more than two-thirds had at least one parent with at least a bachelors degree, while the other third did not have a parent with a degree.

**Table 1:** Profiles of new medical graduates: 2010, 2011 and 2012 batches

**Sample profiles**	**2010**	**2011**	**2012**
Total number of respondents (% of response)	576 (37.2)	872 (59.0)	754 (44.9)
Mean age (SD)	24.3 (1.2)	24.3 (1.4)	24.1 (0.9)
Sex (%)			
• Female	362 (63.1)	516 (61.4)	452 (60.5)
•Male	212 (36.9)	324 (38.6)	295 (39.5)
Mode of admission (%)			
• Normal track*	442 (76.9)	469 (56.0)	520 (69.2)
• Special track**	133 (23.1)	369 (44.0)	232 (30.8)
Location of medical school (%)			
• Bangkok***	345 (65.8)	433 (56.3)	442 (61.5)
• Outside Bangkok	179 (34.2)	336 (43.7)	277 (38.5)
Location of residence during ages 1 to 15 years old (%)			
• Bangkok	240 (42.5)	227 (27.1)	282 (37.7)
• Provincial city	190 (33.7)	345 (41.3)	286 (38.2)
• Non-provincial city	134 (23.8)	264 (31.6)	180 (24.1)
Location of current residence (%)			
• Bangkok	269 (47.2)	247 (29.4)	316 (42.4)
• Provincial city	163 (28.6)	310 (37.0)	244 (32.8)
• Non-provincial city	138 (24.2)	282 (33.6)	185 (24.8)
Location of secondary school (%)			
• Bangkok	306 (53.2)	276 (33.0)	356 (48.0)
• Provincial city	203 (35.3)	434 (52.0)	291 (39.3)
• Non-provincial city	66 (11.5)	125 (15.0)	94 (12.7)
Education profile of parents (%)			
• Neither mother nor father holds a bachelors degree	157 (28.2)	259 (31.4)	209 (27.9)
• One or both parents holds a bachelor degree	400 (71.8)	566 (68.6)	540 (72.1)

Note:

*Normal track includes competitive national entrance examination and direct admission.

**Special track includes Collaborative Project to Increase Production of Rural Doctors (CPIRD), One District One Doctor (ODOD), and other special quota.

***Refers to Bangkok Metropolitan Region including Bangkok and four neighboring provinces of SamutPrakan, SamutSongkham, Nonthaburi and Prathumtani, with more than 12 million population (20% of total).

### Modes of admission and location of secondary school

Modes of admission were significantly related to the location of the secondary school where these graduates studied at grade 12. In 2010, most graduates who were recruited via the normal track came from secondary schools located in Bangkok (65.3%), followed by those located in a provincial city (23.1%). Only 11.6% of graduates came from secondary schools outside a provincial city.

Among graduates recruited through the special track, most of them (76.0%) came from secondary schools in a provincial city; a small proportion (10.5%) came from outside a provincial city. Despite the intention of offering more opportunities for students living in either provincial or non-provincial cities through special track recruitment, a meaningful portion of graduates (13.5%) came from secondary schools in Bangkok. Similar findings emerged across the three batches (Table 2).

**Table 2:** Association between mode of admission and location of secondary school

**Location of secondary schools**	**2010**		**2011**		**2012**	
	**Normal track (%)**	**Special track (%)**	**Normal track (%)**	**Special track (%)**	**Normal track (%)**	**Special track (%)**
Bangkok	288 (65.3)	18 (13.5)	255 (54.6)	20 (5.5)	340 (66.4)	16 (7.0)
Provincial city	102 (23.1)	101 (76.0)	146 (31.3)	286 (78.3)	109 (21.3)	181 (79.4)
Non-provincial city	51 (11.6)	14 (10.5)	66 (14.1)	59 (16.2)	63 (12.3)	31 (13.6)
Total	441 (100.0)	133 (100.0)	467 (100.0)	365 (100.0)	512 (100.0)	228 (100.0)
*P*value	<0.001***		<0.001***		<0.001***	

***, Statistical significance at 99.9% level of confidence.

### Modes of admission and attitudes towards working in rural areas

**Figure 1:**
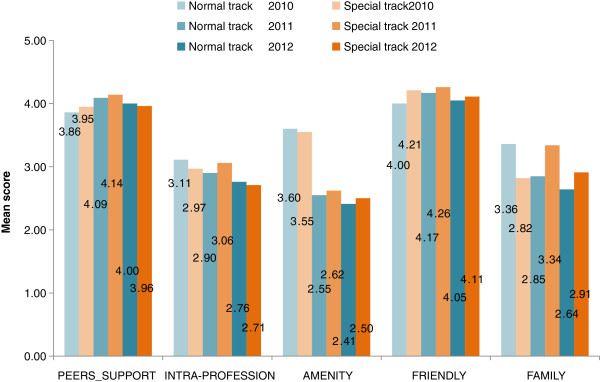
**Attitudes towards rural health services by mode of admission.** PEERS_SUPPORT, peers in rural settings are supportive; INTRA-PROFESSION, workers in rural areas have greater opportunity in meeting professional colleagues; AMENITY, amenities and facilities are available in rural areas; FRIENDLY, people in rural areas are generous and friendly; FAMILY, workers in rural areas can maintain close contact with friends and families.

Across the three batches of graduates, there is a slight difference in rural attitudes between those recruited through the normal track and the special track. Graduates from the special track scored higher on attitude (four out of five) on the impression that people in rural areas are generous and friendly and there is good professional peer support in rural settings. However, on the lack of amenities and facilities in rural areas, and inadequate meetings with professional colleagues they got a lower attitude score, less than 3 out of 5 (Figure 1).

### Modes of admission and intention to fulfil the 3-year compulsory rural service

The univariable analysis in Table 3 shows the association between seven attributes and the intention to fulfil their obligations. Despite all graduates being subject to mandatory rural service, a number of them chose not to adhere to this and instead paid the financial penalty.

**Table 3:** Graduate attributes and intention to fulfil the 3-year mandatory service: a univariable analysis

**Graduate attributes**	**Intention to fulfil the 3-year mandatory service**		
	**2010**	**2011**	**2012**
Sex (%)	*P* = 0.758	*P* = 0.819	*P* = 0.104
• Female	279 (79.7)	439 (87.5)	347 (81.1)
• Male	160 (80.8)	278 (86.9)	212 (76.0)
Mode of admission	*P*< 0.001***	*P*< 0.001***	*P*< 0.001***
• Normal track	323 (76.7)	375 (82.2)	366 (74.1)
• Special track	118 (91.5)	341 (93.7)	193 (90.2)
Location of medical school	*P* = 0.099	P = 0.690	*P* = 0.034*
• Within Bangkok and vicinity	258 (78.7)	365 (85.9)	320 (75.8)
• Outside Bangkok and vicinity	145 (84.8)	285 (86.9)	211 (82.8)
Location of residence during ages 1 to 15 years old (%)	*P* = 0.001**	*P*< 0.001***	*P* = 0.002**
• Bangkok	169 (73.8)	174 (79.1)	192 (71.9)
• Provincial city	161 (88.0)	303 (89.1)	224 (83.6)
• Non-provincial city	104 (81.9)	237 (91.5)	141 (82.5)
Location of current residence (%)	*P* = 0.017*	*P*< 0.001***	*P*< 0.001***
• Bangkok	193 (75.1)	190 (79.2)	218 (72.4)
• Provincial city	134 (85.9)	276 (91.1)	197 (86.0)
• Non-provincial city	109 (83.2)	250 (89.9)	142 (82.1)
Location of secondary school (%)	*P* = 0.001**	*P*< 0.001***	*P*< 0.001***
• Bangkok	223 (76.1)	215 (80.2)	251 (73.8)
• Provincial city	173 (88.7)	390 (91.1)	230 (86.8)
• Non-provincial city	44 (72.1)	107 (88.4)	72 (76.6)
Education profile of parents (%)	*P* = 0.241	*P* = 0.188	*P* = 0.677
• Neither mother nor father holds a bachelors degree	126 (83.4)	228 (89.41)	158 (80.2)
• One or both parents holds a bachelor degree	304 (79.0)	476 (86.08)	401 (78.8)

*, Statistical significance at 95% level of confidence; **, statistical significance at 99% level of confidence; ***, statistical significance at 99.9% level of confidence.

From the analysis, four out of seven attributes have a statistical association with the intention to fulfil the 3-year compulsory rural service. Medical graduates across the three batches who were recruited through the normal track, who spent their childhood life in Bangkok, whose current residence was in Bangkok and whose secondary school was located in Bangkok had a lower intention to fulfil the 3-year compulsory service with statistical significance. It is noted that graduates from medical schools outside Bangkok in the 2012 batch had a higher intention to fulfil their obligations with statistical significance, but this was not the case in the other two studied batches.

Furthermore, when multivariable analysis was conducted using the five attributes having significant influence on the intention to fulfil the 3-years compulsory service, the odds of having the intention to fulfil the 3-year compulsory service among graduates recruited thorough the special track were 2.23-, 2.45-, and 2.46-fold larger than graduates recruited through the normal track in the three batches with statistical significance. Meanwhile the other four attributes showed no significance (Table 4).

**Table 4:** Key attributes and odds ratio of intention to fulfil the 3-year mandatory service: a multivariable logistic regression with marginal effects

	**2010**			**2011**			**2012**		
	**Odds ratio (absolute margins)**^**$**^	**95% Confidence interval**	***P*value**	**Odds ratio (absolute margins)**^**$**^	**95% Confidence interval**	***P*value**	**Odds ratio (absolute margins)**^**$**^	**95% Confidence interval**	***P*value**
Special track recruitment	2.23 (0.12)	1.08-4.59	0.030*	2.45 (0.10)	1.38-4.33	0.002**	2.46 (0.15)**	1.38-4.40	0.002**
Location of medical school outside Bangkok	1.12 (0.02)	0.62-2.03	0.705	0.59 (−0.06)	0.34-1.00	0.050	0.94 (−0.01)	0.57-1.52	0.789
Location of residence during ages 1 to 15 years old outside Bangkok	1.71 (0.08)	0.71-4.13	0.232	1.10 (0.01)	0.47-2.57	0.826	0.98 (−0.00)	0.50-1.91	0.951
Location of current residence outside Bangkok	0.70 (−0.05)	0.26-1.89	0.484	1.73 (0.06)	0.69-4.33	0.244	1.48 (0.06)	0.71-3.09	0.295
Location of secondary school outside Bangkok	1.15 (0.02)	0.54-2.42	0.722	1.16 (0.02)	0.55-2.47	0.699	0.94 (−0.01)	0.53-1.66	0.825

The goodness of fit using the Hosmer and Lemeshow test showed a Pearson Chi-square and its *P*value as follows: 28.15 (*P*value = 0.030), 8.27 (0.94) and 30.26 (0.025) for the 2010, 2011, and 2012 batch, respectively. ^$^Absolute margins are the absolute effects (probabilities) of intention to fulfil the 3-year obligations given the particular variable compared to the probabilities without that variable. For instance, in the 2010 batch, graduates from special track had 12% higher probabilities to fulfil the 3-year obligations than those from the normal track. *, Statistical significance at 95% level of confidence; **, statistical significance at 99% level of confidence.

### Modes of admission and competency

When Principal Component Analysis was applied, the fourteen questions were regrouped into five main domains, namely ‘COMMUNICATE’ (including communication and inter-professional collaboration), ‘PUB_HEALTH’ (including health administration and public health competency), ‘PROCEDURE’ (including management of referral cases, clinical competency, clinical knowledge and clinical skill), ‘MAJ_CLINIC’ (including competencies in internal medicine care, pediatric care, surgical care and general disease care), and ‘OB_GYN’ (including managing difficult labor and obstetric and gynecologic care).

**Figure 2:**
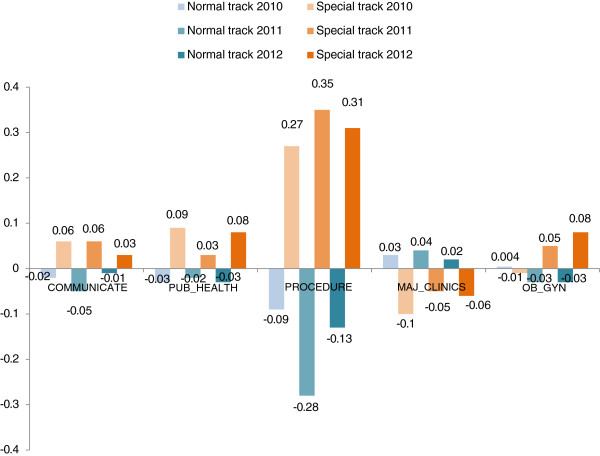
**Composite score for each category of competency, classified by mode of admission.** COMMUNICATE, communication skill; PUB_HEALTH, public health and administrative skill; PROCEDURE, procedural skill; MAJ_CLINIC, clinical knowledge in major subjects; OB_GYN, obstetric and gynecologic skill.

Figure 2 clearly shows that graduates recruited by special track across the three batches had higher scores in COMMUNICATE by showing an average score of 0.06, 0.06, and 0.03 in 2010, 2011, and 2012, respectively. A similar pattern was also observed in PUB_HEALTH (0.09, 0.03, and 0.08), and PROCEDURE (0.27, 0.35, and 0.31 in the three corresponding batches). The level of confidence among graduates recruited by special track in obstetric and gynecologic skill (OB_GYN) also increased between 2011 and 2012. While graduates recruited by special track had higher competency in four domains, those recruited by normal track had higher competencies in major clinical subjects (MAJ_CLINIC) with an average score of 0.03, 0.04, and 0.02, respectively.

## Discussion

The MOPH resignation records indicated that there were increased numbers of doctor who did not complete their rural service obligations. Lower workloads and higher incentives in the private sector were among several main reasons given [17]. The existing financial incentive regimes alone may not be able to overcome stronger pull factors; policymakers need to revisit the question of what are effective interventions for sustaining rural retention.

Graduates from the CPIRD and ODOD programs had slightly better rural attitudes than those from the normal track, albeit medical students from all tracks were exposed to rural health services such as preventive or community medicines, as required by the curriculum, for a few months in their clinical years.

The significantly higher intention to fulfil their compulsory service among graduates from the special track can be explained by a few factors: greater satisfaction when posted in their hometown, in neighboring districts or in provinces which shared the same culture, dialect and social network. Graduates from CPIRD and ODOD were trained in MOPH-affiliated hospitals during clinical years which can provide professional peer support later in their career. This study confirmed previous findings that, after 3 years of mandatory service, 83% of graduates from CPIRD and 52% from the normal track continued in rural practice [21]. Graduates from CPIRD track served for a longer period with a median of 10 years in the public sector; this contrasted with a median of 6.5 years for graduates from the normal track [22,23].

Clinical competencies among CPIRD and ODOD graduates were clearly higher than those from the normal track; this likely came from the greater opportunities to handle cases for students in affiliated MOPH provincial or regional hospitals. By contrast, between normal track medical students and patients, there were layers of first- to third-year residents undertaking their specialty training in university medical faculties; this meant that opportunities to handle cases by medical students were reduced. In addition, patients in affiliated MOPH hospitals presented with more common complaints, like those found in clinical practice after graduation (for example, patients in university hospitals tend to present with more complicated problems, or are referred from elsewhere); they therefore commonly require specialist services, not easily handled by medical students.

Based on the two merits of the special track (higher intention to complete the mandatory 3-year public service, and better clinical competencies) policymakers should expand the proportion of enrolment from CPIRD and ODOD. Unfortunately, the proportion of students recruited through the special track was less than 20% of the total annual medical student enrolment over the past decade, though this had improved recently (30% of new students graduating in the 2013 academic year were recruited through the special track [23,24]).

The ODOD program is more effective at solving shortages than the CPIRD scheme; the districts with chronic shortages are targets for ODOD enrolment and placement upon graduation and ODOD enrolment comes with a longer period of mandatory service. The ODOD design precisely addresses the problem of districts with chronic shortage. Problems have arisen because most students from these remote districts have not been able to pass the special track examination, while medical faculties maintain education standards. Between 2007 and 2009, the balance between the two special track programs saw CPIRD at 63% and ODOD at 37% [23]. The potential merits of ODOD justify seeking to increase the proportion of students recruited through this program.

Higher commitment to rural work among graduates recruited from rural areas was reported from Japan, while Canadian doctors with rural backgrounds had higher interests in rural family practice once they graduated [8-10]. In Australia, students who spent their clinical years in non-traditional medical schools had higher confidence and were better prepared than those who studied in traditional medical schools; this was the result of more hands-on experience and closer patient contact [11].

A few limitations of this study have been identified. Compared with a case–control or cohort design, multiple cross-sectional surveys cannot document causal relationships [25]. It was recognized that the graduates who gathered on the survey date did not represent the whole population; some 20% of total graduates chose to work in non-MOPH hospitals such as military or psychiatric hospitals [26,27] and they therefore did not show up on the survey date. It should be noted that variations in curriculum and pedagogy between universities resulted in variations in competencies. It should be remembered that the intention to work in rural areas is influenced by various contextual environments. Since the recovery from the 1997 economic crisis in early 2000, the private sector’s demand for physicians has grown significantly and contributed to a domestic brain drain of physicians from the public sector [18]. In the context of the upcoming 2015 inauguration of the Association of Southeast Asian Nations Economic Community [28], the growth in economic centers may also stimulate losses of medical professionals from rural areas. The intention to fulfil the mandatory service can be very subjective and unreliable; however, the intention not to fulfil can be very reliable.

### Conclusion and policy recommendations

Evidence suggests that special track recruitment is better than normal track recruitment in terms of the intention to fulfil the mandatory rural service and higher clinical competency. Furthermore, ODOD provides a more precise method of solving chronic shortage in certain districts than CPIRD. Based on this evidence, it is recommended to expand the proportion of recruitment from the special track, while reducing normal track recruitment. Within the special track, efforts should be given to increase the proportion of students recruited through the ODOD program.

### Ethics approval

The National Ethical Review Committee waived ethical clearance as this is a regular monitoring work conducted by the Government, confirmed by ethical review committee: IHRP 47.2/2553 date 28 January 2553 BE (2010 AD). However, informed consent was sought and the protection of confidentiality was strictly followed.

## Abbreviations

CPIRD: Collaborative Project to Increase Production of Rural Doctors; MOPH: Ministry of Public Health; ODOD: One District One Doctor.

## Competing interests

The authors declare that they have no competing interest.

## Authors’ contributions

The study was designed by WP, RS, NT, and VT, TW, NT, and TT reviewed the literature. Data collection and analysis was performed by WP, RS, NT and TT. All authors contributed to drafting and revision and approved the final version of the manuscript.
